# Abundance–activity decoupling in sulfur-cycling bacteria reflects viral infection types in meromictic lakes

**DOI:** 10.1093/ismejo/wrag197

**Published:** 2026-07-31

**Authors:** Jordan R Walker, Natascha S Varona, Bailey A Wallace, Abelardo Aguilar, Molly D O’Beirne, Josef P Werne, Antoni Luque, William P Gilhooly, Alice Bosco-Santos, Cynthia B Silveira

**Affiliations:** Department of Biology, University of Miami, Coral Gables, FL 33146, United States; Department of Biology, University of Miami, Coral Gables, FL 33146, United States; Department of Environmental Science, Policy, and Management, University of California Berkeley, Berkeley, CA 94720, United States; Department of Biology, University of Miami, Coral Gables, FL 33146, United States; Department of Biology, University of Miami, Coral Gables, FL 33146, United States; Department of Geology & Environmental Science, University of Pittsburgh, Pittsburgh, PA 15260, United States; Department of Geology & Environmental Science, University of Pittsburgh, Pittsburgh, PA 15260, United States; Department of Biology, University of Miami, Coral Gables, FL 33146, United States; Department of Physics, University of Miami, Coral Gables, FL 33146, United States; Department of Chemical, Environmental & Materials Engineering, University of Miami, Coral Gables, FL 33146, United States; Department of Earth & Environmental Sciences, Indiana University Indianapolis, Indianapolis, IN 46202, United States; Institute of Earth Sciences, University of Lausanne, Lausanne, 1015, Switzerland; Department of Biology, University of Miami, Coral Gables, FL 33146, United States; Department of Marine Biology and Ecology, Rosenstiel School of Marine, Atmospheric, and Earth Science, University of Miami, Miami, FL 33149, United States

**Keywords:** bacteriophages, green sulfur bacteria, purple sulfur bacteria, sulfate-reducing bacteria, metagenomics, metatranscriptomics, Hi-C, anoxygenic photosynthesis, viral metabolic genes

## Abstract

Meromictic lakes serve as analogs of redox-stratified ancient oceans with well-mixed surface waters and anoxic bottoms. In sulfide-rich lakes, purple and green sulfur bacteria (PSB, GSB) dominate the anoxic zones where light penetrates, and their biosignatures can guide interpretations of geologic records. Although PSB and GSB biosignatures indicate presence, they do not directly reflect the community composition of modern analog lakes, posing a challenge for interpretation. Here, we investigate this decoupling by integrating metagenomics, metatranscriptomics, and metaHi-C virus–host linkages with the geochemical profiles of three meromictic lakes. In the phototrophic microbial plates, PSB transcriptional activity far exceeded their abundance (73% of total microbial community activity versus 30% of abundance), whereas GSBs displayed the opposite pattern. Concurrently, PSBs were exclusively associated with temperate viruses and GSBs were targeted by lytic infections. Sulfate-reducing bacteria and viruses encoding genes for sulfate reduction were most active where sulfide concentration was lowest. These results reveal that viral replication strategies are associated with the decoupling between abundance and activity in anoxygenic phototrophs and sulfate reducers. These relationships could accelerate sulfur regeneration, contribute to sustaining phototrophy, and ultimately reflect in the lake’s bulk biosignatures.

## Introduction

In the Precambrian, prior to the oxygenation of Earth’s atmosphere (>2.4 Ga), anoxygenic photosynthetic bacteria were major contributors to marine primary production [1, 2]. During the Paleozoic, their contribution became context-dependent, expanding during transient episodes of oceanic oxygen limitation, particularly in the Early Paleozoic (540–470 Ma) and Devonian (420–360 Ma) oceans [3–7]. Today, anaerobic photoautotrophs persist as purple and green sulfur bacteria (PSB and GSB), which can dominate illuminated anoxic environments such as meromictic lakes, euxinic and ferruginous basins, and permanently stratified coastal waters [8–12]. Biosignatures (i.e. metabolic outputs) produced by PSB and GSB in modern redox-stratified systems provide a powerful window into anoxygenic primary production [5, 13, 14]. In this context, meromictic lakes have been extensively used to investigate past anoxic environments, to inform interpretations of anoxic settings beyond Earth, and to anticipate future ocean deoxygenation [5, 9, 15–18]. These lacustrine systems are stratified into three distinct layers: an oxygenated seasonally mixed surface layer (mixolimnion), an intermediate chemocline characterized by steep redox and chemical gradients, and a stable anoxic bottom layer (monimolimnion). The chemocline, where light penetrates sulfide-rich waters, provides a unique niche for anoxygenic photoautotrophs to form a dense microbial plate [18–20]. Thus, meromictic lakes can be used to investigate the processes that control biosignature production by PSB and GSB, and their preservation in the geological record.

In illuminated anoxic environments, PSB and GSB typically oxidize reduced sulfur compounds, most commonly sulfide, to fuel photosynthesis [11, 21]. In these settings, they are metabolically coupled to sulfate-reducing bacteria (SRB) through microbial sulfur cycling. Sulfide produced by SRB helps to fuel anoxygenic photosynthesis by PSB and GSB, whereas organic carbon and intermediate sulfur compounds produced by phototrophic sulfur bacteria support heterotrophic metabolisms, including sulfate reduction [22, 23]. This tight coupling establishes steep redox gradients, linking sulfur oxidation and reduction within chemoclines [20, 24]. Sulfur-cycling bacteria, such as SRB, also leave diagnostic biosignatures in the geological record, most notably distinct sulfur isotopic fractionations that extend back >3 billion years [25–27].

Despite the extensive use of PSB and GSB biosignatures to reconstruct anoxic environments, a growing body of evidence indicates that biomarker abundance and carbon and sulfur isotopic ratios are often decoupled from the abundance and composition of sulfur bacterial communities [5, 28, 29]. Bottom–up controls, such as light and sulfide availability, have been insufficient to explain this decoupling [16, 20, 30–33]. One potential, yet underexplored, explanation for this decoupling is viral modulation of host metabolism. Viruses of bacteria can establish lytic (virulent) infections that kill the host cell, or long-term interactions such as chronic and lysogenic infections, in which the virus replicates without killing the host or integrates into the bacterial genome, respectively. In all types of interactions, the expression of viral-encoded metabolic genes can alter bacterial cell physiology [34–37]. Viral infection can also expand host metabolic capabilities via lateral gene transfers [38, 39] and changes in overall gene expression profiles [40–42]. Recent observations in meromictic lakes include the identification of viruses encoding metabolic genes involved in carbon fixation, sulfur cycling, and pigment production, as well as transitions in viral replication strategies, as indicated by viral abundance, virus-to-microbe ratios, and viral transcriptional activity [17, 18, 43]. Therefore, viral infections may represent a mechanism that decouples abundance and metabolism in sulfur-metabolizing bacteria and, consequently, their biosignatures.

Previous studies support the role of viruses in altering microbial community structure and metabolism in meromictic systems, however, viral interactions with sulfur-metabolizing bacteria remain poorly constrained. In the present study, we aimed to assess the relative importance of lytic versus lysogenic infection strategies, link viruses to their hosts, and quantify the expression of viral-encoded genes with the potential to influence specific pathways of sulfur cycling and carbon fixation. To accomplish these aims, we integrated metatranscriptomic, metagenomic, and proximity ligation (metaHi-C) approaches to study the relationship between the activity of sulfur-metabolizing bacteria, viral replication, and gene expression in three meromictic lakes with sulfide concentrations ranging from trace (0.006 mM) to high (25.39 mM).

## Materials and Methods

### Sampling and geochemical data

**Figure 1:**
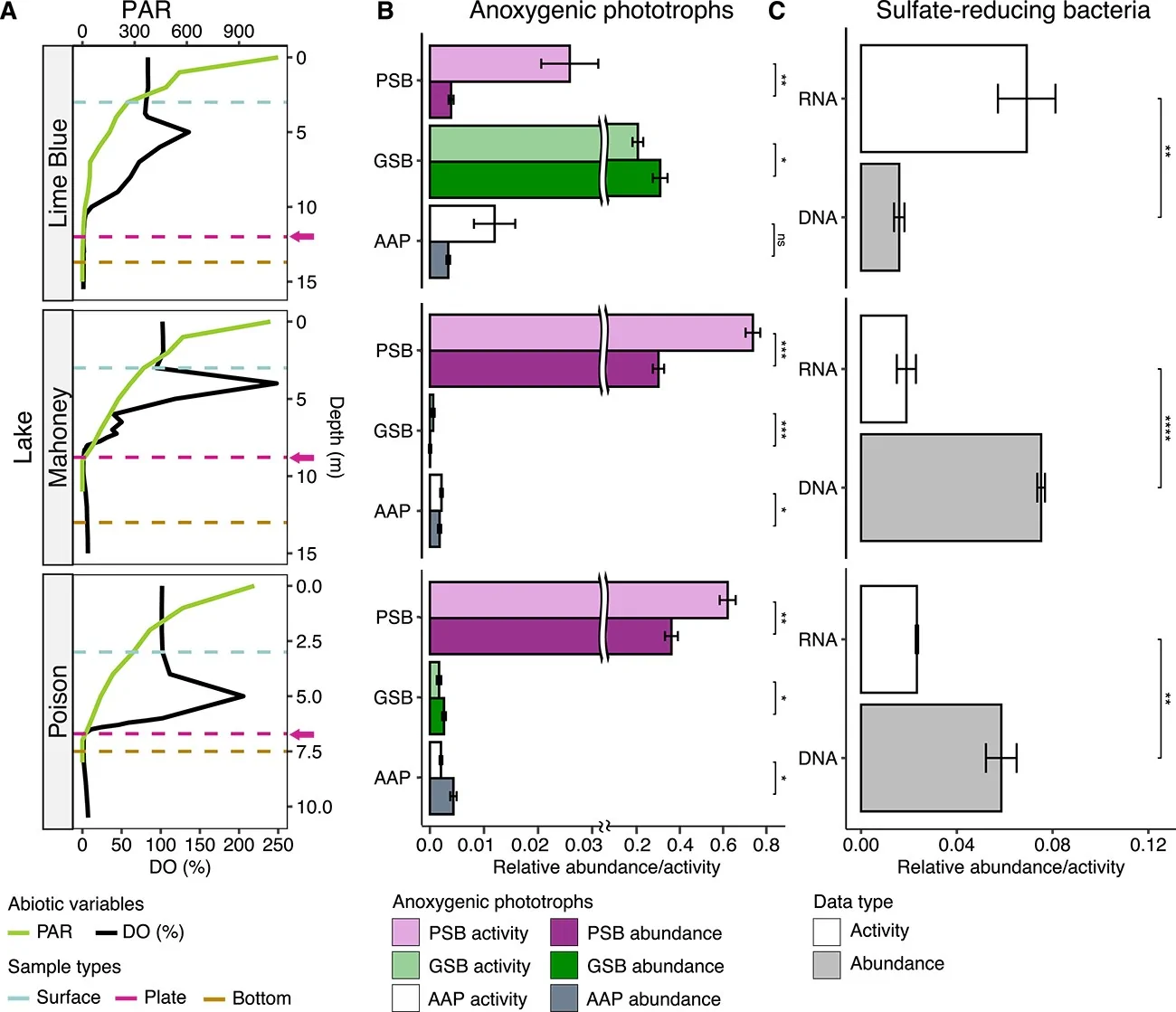
Depth profiles and distribution of anoxygenic phototrophs and sulfate reducers across the three study lakes. (A) Lake vertical profiles indicating sampling depths (dotted lines), dissolved oxygen (%), and photosynthetically active radiation (PAR) for each lake (lake names shown on the left). Dotted horizontal lines indicate the depths at which microbial communities were sampled, including the surface, the microbial plate (arrows), and the bottom layers. (B) Abundance and activity of anoxygenic phototrophs in microbial plate samples. Anoxygenic phototrophs are grouped as PSBs, GSBs, or aerobic anoxygenic phototrophs. (C) Abundance and activity of sulfate-reducing bacteria in microbial plate samples. Values are displayed as the mean and standard error across replicate samples per depth. Results of Tukey’s tests comparing abundance to activity are denoted by ns, not significant, ^*^ ≤ 0.05, ^**^ ≤ 0.01, ^***^ ≤ 0.001, ^****^ ≤ 0.0001.

The three lakes, Mahoney Lake (British Columbia, Canada), Poison Lake (Washington, USA), and Lime Blue Lake (Washington, USA), were sampled between 19 July and 25 July, 2023. Briefly, water column temperature, dissolved oxygen (DO), specific conductivity, and pH were measured using a YSI ProDSS multiparameter probe (YSI Incorporated, Yellow Springs, OH, USA) and photosynthetically active radiation (PAR) was measured using a spherical underwater quantum sensor coupled to a data logger (LI-193 and LI-1500; LI-COR Environmental, Lincoln, NE, USA) (Supplementary Table S1). Water samples for geochemical and microbiological analyses were collected from three discrete depths in five replicates (surface, microbial plate, and bottom) using a peristaltic pump (Electra; Proactive Environmental Products, Bradenton, FL, USA) coupled to a custom-designed lateral intake to minimize vertical mixing (Supplementary Table S2). Sampling depths were: 3.0, 8.78, and 13.0 m for Mahoney Lake (MAH); 3.0, 6.7, and 7.5 m for Poison Lake (POI); and 3.0, 12.0, and 13.7 m for Lime Blue Lake (LB) (Fig. 1A, Supplementary Table S2). The depth of the microbial plate was determined by measuring DO and sampling at 10–5 cm intervals until a change in water coloration was observed. Dissolved sulfide concentrations were measured in 5 ml samples obtained from the chemoclines of the three lakes, fixed with 0.05 M Zn-acetate (Zn(CH_3_COO)_2_•2H_2_O) solution at a 1:2 ratio immediately after sampling, and quantified photometrically using the methylene blue method [44].

Samples for total nucleic acid extractions were collected in replicate using 0.22 μm polyethersulfone (PES) Sterivex filters (Millipore-Sigma, Burlington, MA). Five replicate samples were collected per lake and depth for DNA extraction and five for RNA extraction, for a total of 90 Sterivex filters. Filtered volumes varied with microbial density at each lake depth due to clogging; volumes are reported in Supplementary Table S2. Immediately after collection, 1 ml of RNAlater (Invitrogen, Carlsbad, CA) was added to Sterivex filters for metatranscriptomic analyses. Filters were flash-frozen in liquid nitrogen and stored in dry shippers until their return to the University of Miami laboratory, where they were stored at −80°C. An additional 3 ml of microbial plate water from each lake was filtered through 0.2 μm, 25 mm cellulose nitrate filters (cat #10400106, Whatman, Milwaukee, USA) for metaHi-C cross-linking and library preparation. These filters were also flash-frozen in liquid nitrogen immediately after collection and stored at −80°C until processing.

### Library preparation and sequencing

Total DNA was extracted from the 45 metagenomic samples using a modified Nucleospin Tissue Kit (Macherey-Nagel, Duren, Germany). The protocol was modified by adding 360 μl of T1 buffer and 50 μl of Proteinase K directly to the Sterivex filters, followed by overnight incubation at 55°C. The following day, 400 μl of B3 buffer was added to each Sterivex, and samples were incubated at 70°C for 30 min. The lysate was recovered from the Sterivex filters and mixed with 420 μl of 100% ethanol. The resulting mixture was loaded onto silica spin columns in 600 μl increments and centrifuged at 11 000 × *g* for 1 min. This step was repeated until the entire sample volume had passed through the column. Washing steps were performed according to the manufacturer’s instructions. DNA was eluted in 50–100 μl of molecular-grade water. DNA concentrations were quantified using a Qubit 2.0 Fluorometer (Life Technologies, Carlsbad, CA, USA). For samples with low DNA yield or large elution volumes, DNA was concentrated by ethanol precipitation through the addition of 0.1× the elution volume of 3 M sodium acetate, 2.5× the elution volume of ice cold 100% ethanol, and 1 μl of glycogen, followed by incubation at −20°C for 3 h. Samples were centrifuged at 4°C for 30 min at 21 130 × *g*, after which the supernatant was removed. The DNA pellet was washed with 500 μl of 75% ethanol and centrifuged at 4°C for 10 min at 21 130 × *g*. This wash step was repeated, after which the pellet was briefly spin-dried (10 s) and resuspended in 50 μl of molecular-grade water. DNA extracts were stored at −80°C and shipped to the DOE Joint Genome Institute (JGI) for sequencing. Plate-based DNA library preparation for sequencing was performed on the Hamilton Vantage robotic liquid handling system using Kapa Biosystems HyperPrep library preparation kit (Roche). Genomic sample DNA was sheared to 409 bp using a Covaris LE220 focused-ultrasonicator, fragments were size-selected using TotalPure NGS beads (Omega Bio-tek), and fragments were end-repaired, A-tailed, and ligated with Illumina-compatible unique dual-index sequencing adaptors (Perkin Elmer). Fragments were sequenced using a NovaSeq X System (Illumina), yielding a total of 5 668 261 550 paired-end 2 × 151-bp reads (Supplementary Table S3).

Metatranscriptomes were sequenced and published previously and re-analyzed here in combination with newly generated metagenomes and metaHi-C data [18]. Briefly, metatranscriptomic samples were processed using a modified DNeasy PowerWater Sterivex kit (Qiagen, Germantown, MD, USA) following previously published methods [45]. Fourteen samples failed RNA extraction; for two of these, RNA was extracted from unfiltered flash-frozen water samples using a chloroform/TRIzol reagent (Invitrogen, Waltham, MA, USA). Following treatment with TURBO DNase (Thermo Fisher Scientific, Waltham, MA, USA) and rRNA depletion using QIAseq FastSelect − rRNA 5S/16S/23S kit (Qiagen, Germantown, MD, USA), cDNA libraries were constructed with NEBNext Ultra II RNA Library Preparation Kit for Illumina and sequenced using a NovaSeq System (Illumina), generating a total of 766 883 916 paired-end 2 × 150 bp reads (Supplementary Table S4).

Hi-C libraries were prepared using the ProxiMeta Hi-C v4.0 Kit (Phase Genomics, Seattle, USA). Briefly, filters were cross-linked with formaldehyde and digested with the restriction enzymes Sau3AI and MlucI. DNA fragments were proximity-ligated with biotinylated nucleotides and purified with streptavidin beads prior to addition of indexed primers from the ProxiMeta Hi-C kit. Hi-C libraries were sequenced on a NovaSeq System, producing ~160 M paired-end 2 × 150 bp reads. Hi-C library preparation failed for Lime Blue Lake microbial plate samples; therefore, only Poison Lake and Mahoney Lake chemocline samples yielded Hi-C data.

### Genome binning

Metagenomic reads were processed according to JGI’s standard metagenome workflow [46]. This pipeline includes quality control filtering (bbduk.sh parameters maq = 3.0,0 trimq = 0.0 qtrim = r maxns = 3 minlen = 51 minlenfraction = 0.33 k = 31 hdist = 1), adapter and artifact trimming (bbduk.sh parameters ktrim = r minlen = 51 minlenfraction = 0.33 mink = 11 tbo tpe rcomp = f k = 23 hdist = 1 hdist2 = 1 ftm = 5 pratio = G,C plen = 20), masking of common laboratory and microbial contaminants (bbmap.sh parameters deterministic quickmatch k = 13 idtag = t qtrim = rl trimq = 10 untrim minid = .95 idfilter = .95 maxindel = 3 minhits = 2 bw = 12 bwr = 0.16 maxsites2 = 10 tipsearch = 0), and read error correction to attempt to correct low coverage sequencing errors (bbcms.sh parameters mincount = 2 and highcountfraction = 0.6) using the BBTools Software package version 39.03 [47] (Supplementary Table S3). Assembly of contigs and scaffolds was performed with metaSPAdes (version 3.15.2; parameters -m 2000 --only-assembler -k 33,55,77,99 127 --meta) (Supplementary Table S3), reads were mapped using BBMap (parameters ambiguous = random), and scaffolds were binned into metagenome-assembled genomes (MAGs) with MetaBat2 (version 2.15) using standard parameters [47–49]. MAG completeness and contamination was estimated using CheckM 2 (version 2.15) [50]. MAGs with >50% completion and containing <10% contamination were retained for further analysis in accordance with MIMAG standards for medium- and high-quality draft genomes (*N* = 1430) [51]. Metatranscriptomic reads were quality-controlled and assembled using BBDuk (version 38) and metaSPAdes (version 3.15.5), as previously described [18]. Hi-C reads were preprocessed to cut reads at restriction enzyme sites using hicstuff’s (version 3.2.4) digest function [52]. Hi-C reads were combined with their corresponding scaffolds from the metagenomes described above and binned using Metator version 1.3.10 [53]. This resulted in five separate Hi-C binning sets for both Mahoney and Poison microbial plates, one for each replicate metagenome. Viral sequences (identified in the following section) were removed to yield the final set of prokaryotic MAGs for downstream analyses. CRISPR spacers were identified in these MAGs using MinCED version 0.4.2 [54]. Metagenome and Hi-C-derived MAGs were dereplicated using dRep version 3.5.0 with standard settings [55]. Open reading frames were predicted with Prodigal (version 2.6.3), taxonomic assignment with GTDB-tk (version 2.4.1), antiphage systems with defensefinder (version 2.0.1), and protein annotation of translated amino acid sequences with MetaCerberus (version 1.2.1) [56–59] (Supplementary Table S3).

### Identification of viral genomes

Viral sequences were identified among the metagenomic scaffolds, metatranscriptomic contigs, and quality-filtered metagenomic and Hi-C MAGs using geNomad (version 1.8.0) and VIBRANT (version 1.2.1) [60, 61]. All sequences predicted as viral by geNomad and/or VIBRANT were combined and filtered to remove those shorter than 2.5 kbp. The remaining viral sequences were clustered into viral Operational Taxonomic Units (vOTUs) using the anicalc and aniclust scripts in CheckV (version 1.0.3) with an average nucleotide identity threshold of 95% and a minimum coverage of 85%, retaining the longest sequences, following minimum information about an uncultivated virus genome (MIUVIG) standards [62, 63]. CheckV was used to exclude representative vOTU sequences (hereafter simply referred to as viruses) lacking viral hallmark genes and to remove host regions flanking viruses identified as prophages. MetaCerberus with prodigal-gv was used for viral gene annotation [57, 58, 60]. Putative temperate viruses, regardless of completion, were identified based on the genomic potential of the virus to integrate using VIBRANT parameters for prophage identification and keywords in the MetaCerberus annotations, including integrase, recombinase, transposase, excisionase, Cro/C1, phage (anti)repressor, PHROG or VOG-derived ParAB, and phage regulatory protein CII. Integrated prophages were identified using a combination of three different methods: (i) viral OTUs that contained member sequences identified as prophages with host flanking regions during viral identification by geNomad or VIBRANT, (ii) identification of prophages using CheckV, and (iii) BLASTn (version 2.16.0) alignment of predereplicated MAGs to viruses with at least 95% identity, 100% coverage of the virus, and 500-bp flanking regions on each end of each viral sequence [64]. The methods used here may lead to an underestimation in predicted temperate viruses due to the difficulty in identifying integration genes or flanking regions in incomplete viral genomes. Thus, viruses not labeled as temperate are considered putatively lytic, herein referred to as lytic viruses.

Putative viral hosts were identified in four ways: (i) alignment of CRISPR spacers from dereplicated MAGs to viruses with 100% coverage, less than two mismatches, and a minimum length of 20 bp; (ii) prophages integrated into MAGs; (iii) host prediction using iPHoP with a minimum confidence score of 0.95; and (iv) sequences identified as viral using geNomad or VIBRANT within Hi-C MAGs created using MetaTor [64–67]. MetaTor creates MAGs consisting of subnetworks of contigs enriched in Hi-C contacts by unambiguously aligning Hi-C reads to contigs, normalizing read coverage to the geometric mean of coverage of all contigs, and performing 100 iterations of Louvain clustering with contigs as nodes and normalized coverage as weight values [53]. All unique virus–host interactions observed were compiled into a bipartite matrix and visualized in Cytoscape v. 3.10.3, with hosts classified at the order level and viruses classified according to lifestyle [68]. We employed Virsorter2 to further inspect PSB MAGs for potential prophages that may have been missed in the pipeline utilized for metagenomes, metatranscriptomes, and Hi-C MAGs [69].

### Calculation of relative abundance and activity

Metagenomic and metatranscriptomic reads were mapped to dereplicated MAGs and viruses using BBMap with standard settings. Resulting SAM files were converted to BAM format, sorted, and indexed using samtools (version 1.21) [70]. The number of reads or transcripts mapping to each contig was quantified using CoverM (version 0.7.0) [71]. Fractional abundances and transcription of MAGs and viruses were calculated using the equation:


$$f(i)=\frac{r(i)}{T(j)}\times \frac{L(mean)}{L(i)}$$

where *r(i)* is the number of reads/transcripts mapped to a MAG or vOTU, *T(j)* is the total number of reads/transcripts in a metagenome/metatranscriptome, *L(mean)* is the mean contig or virus length, and *L(i)* is the length of a MAG or vOTU. The relative abundances and transcription were calculated as *f(i)* divided by the sum of all *f(i)* within a metagenome/metatranscriptome. Statistical analysis and visualization were done in RStudio (version 2025.05.0 + 496) and R (version 4.4.2) [72, 73]. Statistical tests were performed using the package rstatix (version 0.7.2) [74]. Data visualization was performed using ggplot2 (version 3.5.2), ggpubr (version 0.6.1), and ggsci (version 3.2.0) [75–77].

### Phylogenetic analysis of viral metabolic genes

Viral metabolic genes with the same predicted function were aligned in MAFFT (version 7). Gene-specific profile hidden Markov models (HMMs) were constructed with HMMER 3.4  hmmbuild [78, 79]. HMM profile scanning was selected to enable sensitive detection of cellular homologs from viral query sequences. Each profile was searched with  hmmsearch  against Swiss-Prot and NCBI RefSeq. Hits were retained if both the full-sequence and best-domain e-values satisfied e < 1 × 10^−10^, reducing false positives from multi-domain proteins where only a nonhomologous region produces a spurious signal. The same profiles were used to identify homologs among MAGs from the lake dataset. Viral metabolic genes and all database and MAG hits were pooled into per-gene datasets. Exact duplicates were removed, and database hits were filtered to known orders of PSB, GSB, or SRB. Multiple sequence alignments of viral metabolic genes, MAG homologs, and filtered database hits were generated with MAFFT. For datasets below 2000 sequences,  --auto  mode was used, and, for larger datasets, a partition-tree progressive strategy, --parttree. Poorly aligned columns were removed with trimAl [80]. Maximum-likelihood trees were inferred with IQ-TREE 2 [81]. The best-fitting substitution model for each gene was identified by ModelFinder using the Bayesian information criterion [82]. Branch support was assessed with 1000 ultrafast bootstrap replicates. Trees were visualized in the Interactive Tree of Life (version 7.6) [83]. Nodes were collapsed to emphasize the closest relatives to viral sequences found in this study. Three-dimensional protein structures of viral metabolic genes and their closest PSB, GSB, or SRB genes were generated on the AlphaFold Server [84]. Visualization and structure comparison were performed in ChimeraX (version 1.12) using the matchmaker function [85].

## Results

### Environmental gradients and microbial plate characterization

Dissolved oxygen concentrations peaked at ~5 m depth in all three lakes, followed by a sharp transition to anoxia at ~7–8 m at Mahoney and Poison Lakes and a more gradual decline to anoxic conditions at ~10 m at Lime Blue Lake (Fig. 1). Photosynthetically active radiation (PAR) decreased steadily with depth and approached zero at the microbial plate sampling depths in all lakes (Fig. 1A). Depleted dissolved oxygen concentrations, lower PAR values, and visible color changes observed on 0.45 μm filters were used to define the sampling depth of the microbial plate. Based on these criteria, the microbial plates were defined at 8.78 m in Mahoney Lake, 6.7 m in Poison Lake, and 12 m in Lime Blue Lake. Sulfide concentration varied by several orders of magnitude across the sampled microbial plates, ranging from 0.006 mM in Lime Blue Lake to 0.831 mM in Poison Lake, and reaching a maximum of 25.389 mM in Mahoney Lake.

### Abundance and transcriptional activity of anoxygenic phototrophs

Assembly and binning of metagenomic and Hi-C reads resulted in 580 high or medium-quality, unique MAGs (Supplementary Table S5). Of these, 513 were classified as Bacteria and 67 as Archaea (Supplementary Table S5). Thirteen MAGs were classified as potential anoxygenic phototrophs based on the presence of genes encoding anoxygenic photosynthetic reaction centers, including *pufLM* and photosystem P840. Two anoxygenic phototroph MAGs were classified as PSBs within the order *Chromatiales*, genera *Chromatium* and *Thiohalocapsa*. The *Thiohalocapsa* MAG was the most abundant MAG in the microbial plates of Mahoney and Poison Lakes, accounting for 30.1% and 36.1% of the microbial community, respectively, and 73.5% and 62.0% of the community transcriptional activity (herein after referred to as activity) (Fig. 1B). In contrast, the *Chromatium* MAG accounted for <1% of the community and activity in all samples, except in Lime Blue’s microbial plate, where it accounted for 0.4% of the abundance, but its activity reached 2.0%. Across all three microbial plates, PSB exhibited significantly higher relative activity than what would be predicted from their abundance (Tukey’s test, *P*-value <.05).

Four anoxygenic phototrophic MAGs were classified as GSBs, including three belonging to the genus *Chlorobium* and one *Prosthecochloris*. One *Chlorobium* MAG dominated the Lime Blue microbial plate, accounting for 29.3% of the community composition and 19.6% of the activity (Fig. 1B). The remaining GSB MAGs each contributed <2% and 1% of the abundance and activity on the Lime Blue microbial plate, respectively. The GSBs’ activity was significantly lower than what would be predicted by their abundance in Lime Blue and Poison Lake microbial plates (Fig. 1B; Tukey’s test, *P*-value <.05) but higher in Mahoney Lake, where both activity and abundance were extremely low (<0.006% and <0.0004%, respectively).

The remaining seven anoxygenic phototrophic MAGs belonged to the orders *Burkholderiales, Caulobacterales, Rhodobacterales, Sphingomonadales*, and *Chthoniobacterales*. Except for *Chthoniobacterales*, all these orders contain known aerobic anoxygenic phototrophs. Consistent with their aerobic nature, aerobic anoxygenic phototrophs had the highest abundances and activities in Mahoney and Poison surface waters (Supplementary Fig. S1A). Together, aerobic anoxygenic phototrophs accounted for 1.2% or less of the community composition and activity in all microbial plates (Fig. 1B).

MAGs classified as SRBs [orders *Desulfobacterales* (*n* = 11), *Desulfobulbales* (*n* = 7), and *Desulfatiglandales* (*n* = 10)] comprised a smaller fraction of the microbial community abundance and activity compared to the phototrophs (Fig. 1B and C, note differences in scale). SRBs were significantly more active than their abundance would predict in the Lime Blue microbial plate (6.9% and 1.6%, respectively), whereas the opposite was observed in Mahoney and Poison Lakes (Tukey’s test, *P*-value <.05 for all comparisons, Fig. 1). SRBs were most abundant in bottom water samples, where abundance and activity were often coupled (Supplementary Fig. S1B).

### Viral community abundance and transcriptional activity

**Figure 2:**
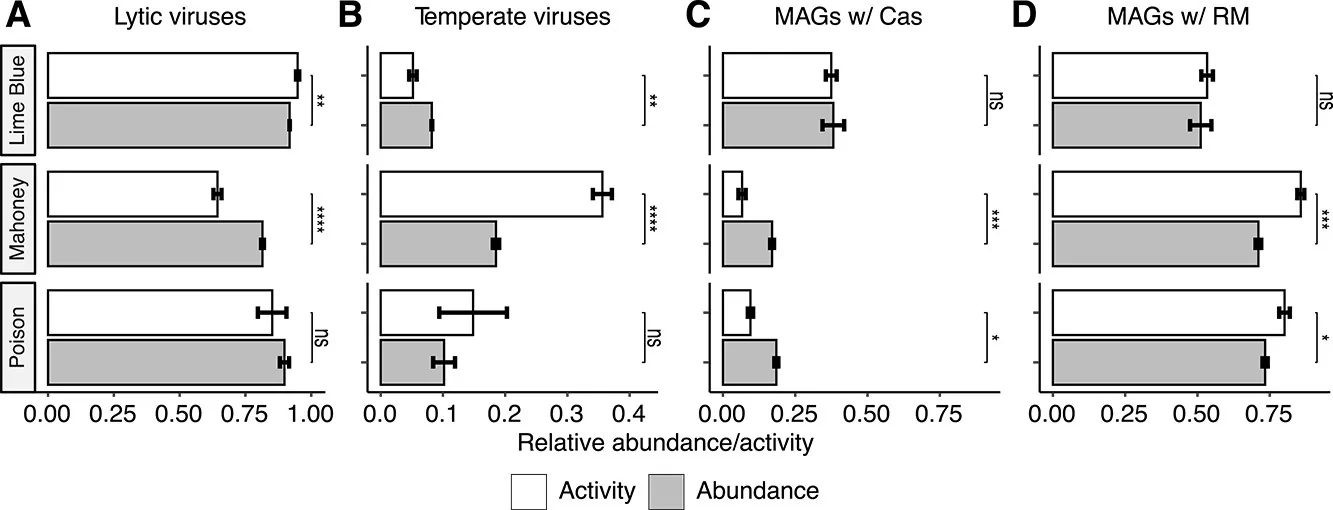
Abundance and activity of lytic and temperate viruses and hosts with defense systems in the microbial plates. Abundance is indicated by gray bars and activity by white bars. (A) Viruses classified as lytic, (B) viruses classified as temperate, including prophages, (C) MAGs with genes involved in Cas defense systems, and (D) MAGs with genes annotated as RM systems. Values are displayed as the mean and standard error across replicates. Results of Tukey’s tests comparing abundance to activity are denoted by ns, not significant, ^*^ ≤ 0.05, ^**^ ≤ 0.01, ^***^ ≤ 0.001, ^****^ ≤ 0.0001.

A total of 204 280 unique sequences were classified as putative viral genomes or genome fragments (herein viruses) >2.5 kbp (189 279 by geNomad and 98 544 by VIBRANT, with 83 543 identified by both viral prediction tools). Clustering and quality control of the viral contigs yielded 44 392 viruses, 4269 of which were classified as temperate; among these, 1895 were identified as integrated prophages (Supplementary Table S6). Lytic virus activity was significantly higher than their abundance in all samples except for the microbial plates of Mahoney and Poison (Tukey’s test, *P*-value <.05, Fig. 2A, Supplementary Fig. S2A). At these two microbial plates, temperate viral activity was the highest among all samples (35.6% and 14.9% of the total viral activity, respectively, Fig. 2B, Supplementary Fig. S2B), and in Poison Lake, it was higher than temperate virus abundance (Tukey’s test, *P*-value <.05; in Mahoney Lake, the difference was not significant).

Among all prokaryotic MAGs in the dataset, 74% carried phage defense systems, including all the PSB and GSB MAGs. MAGs encoding Cas systems were more abundant in the Lime Blue microbial plate, accounting for 38.2% and 37.5% of the community abundance and activity, respectively (Fig. 2C). In Mahoney and Poison Lakes, MAGs with Cas systems were less abundant (18.3% and 17.0%, respectively) and displayed lower activity relative to their abundances (Fig. 2C). MAGs containing restriction–modification (RM) systems showed the opposite trend, with a higher overall abundance and activity in Poison and Mahoney relative to Lime Blue, and higher activity relative to abundances in Mahoney and Poison Lakes (Tukey’s test, *P*-value <.05, Fig. 2D). The only other sample in which MAGs with Cas and RM systems displayed high activity relative to abundance was the bottom layer of Poison Lake (Supplementary Fig. S2D).

### Virus–host interaction network

Four virus–host linkage methods (MetaHi-C, CRISPR spacer matches, prophage matches, and iPHoP) identified 1013 unique putative associations between viruses and MAGs (Supplementary Fig. S3A, Supplementary Table S7). The predicted virus–host network consisted of 970 viruses and 247 MAGs, where 43 viruses interacted with more than one MAG (Supplementary Fig. S3, Supplemental Table S7). Despite using only two Hi-C samples, Hi-C links accounted for 63.6% of total interactions; of the Hi-C links, 2% (19 of 674) were supported by CRISPR spacers, MAG prophages, or iPHoP (Supplementary Fig. S3B and C). The virus–host interaction network was highly fragmented, displaying 221 connecting groups with an average of 1.7 neighbors (Supplementary Fig. S3). This fragmentation likely reflects the network’s representation of all virus–host interactions from different, likely independent, sample types (surface, microbial plate, and bottom waters), as well as false-negative linkages across groups.

**Figure 3:**
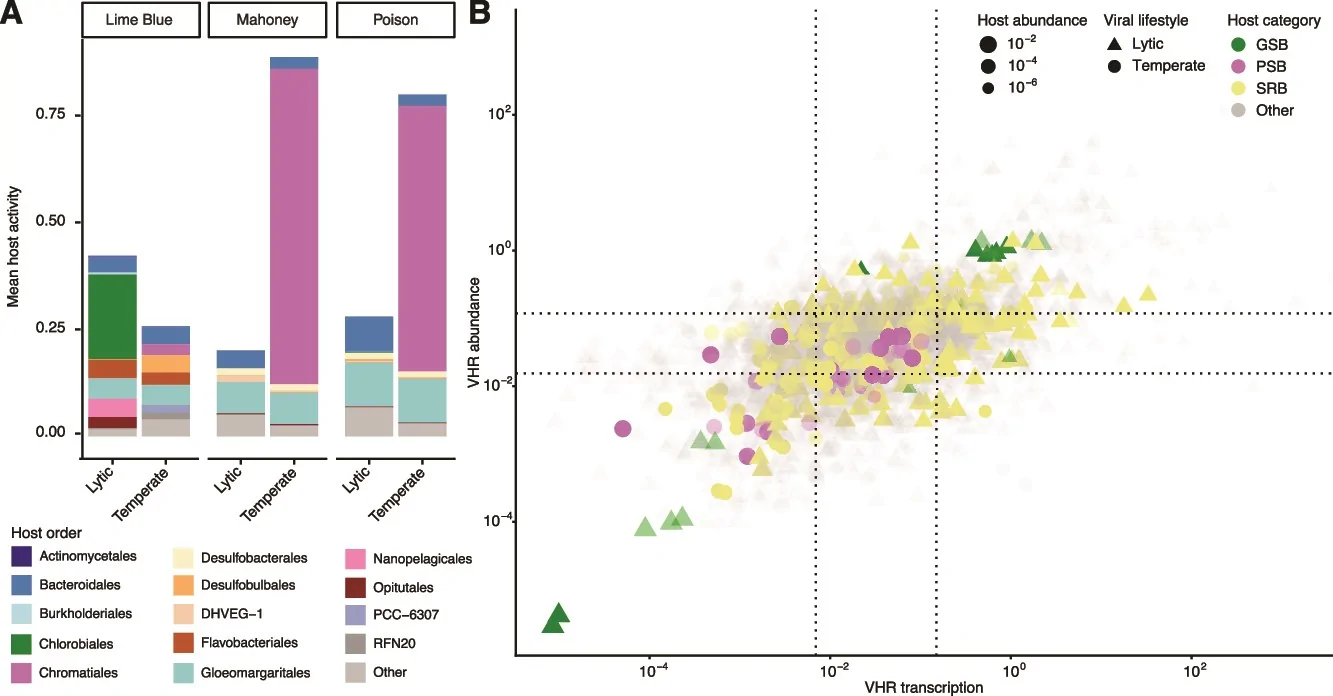
Virus–host dynamics of meromictic lakes. (A) The mean activity of hosts associated with lytic and temperate viruses. Bars represent the taxonomic classification of the host at the order level. (B) Relationship between the virus-to-host ratio (VHR) calculated from relative abundances (*Y*-axis) and relative activity (*X*-axis) across all samples. Opaque points represent viruses with connections to PSB, GSB, or SRB in the microbial plates. Transparent points include all other bacteria and PSB, GSB, or SRB virus–host associations on the surface or bottom of the lakes. Triangles represent putatively lytic vOTUs, and circles indicate temperate vOTUs. Point sizes are scaled to the log-transformed host relative abundance.

In the microbial plates of Mahoney and Poison Lakes, only temperate viruses interacted with the most active and abundant PSB genus, *Thiohalocapsa* (Fig. 3A, Supplementary Fig. S3, Supplementary Table S7). By contrast, all GSBs, including the most dominant in Lime Blue, *Chlorobium*, were associated exclusively with putatively lytic viruses (Fig. 3A, Supplementary Fig. S3, Supplementary Table S7). Virus-to-host ratios (VHRs) were calculated from metagenomes and transcriptomes to indicate virus replication and activity relative to their hosts (Fig. 3). Most of the virus–host pairs outside of the interquartile ranges (dotted lines, *n* = 797, Fig. 3B) fell into the upper-right quadrant (high abundance and activity relative to host, *n* = 351, 44%) or the lower-left quadrant (low abundance and activity relative to host, *n* = 341, 43%). Temperate viruses were enriched in the lower-left quadrant (37%) compared to the upper-right quadrant (18%). PSB–virus pairs fell exclusively within the interquartile range or in the lower-left quadrant, which is characterized by relatively low lytic viral activity and is consistent with temperate viral infections. In contrast, GSB–virus pairs varied between upper and lower quartiles. The most dominant GSB in the chemocline of Lime Blue, *Chlorobium*, exhibited the lowest VHR in abundance and activity across the dataset, which could result from a relatively lower frequency of infection given the large host population (Fig. 3A). Another GSB, displaying 1.5% abundance in the bottom and 0.3% in the chemocline, was associated with one of the most abundant and active viruses, falling in the upper-right quadrant (Fig. 3A). SRBs were associated with a diversity of lytic and temperate viruses, resulting in a broad range of VHRs, indicating high variability in viral activity and replication.

### Metabolic genes encoded by viruses

**Figure 4:**
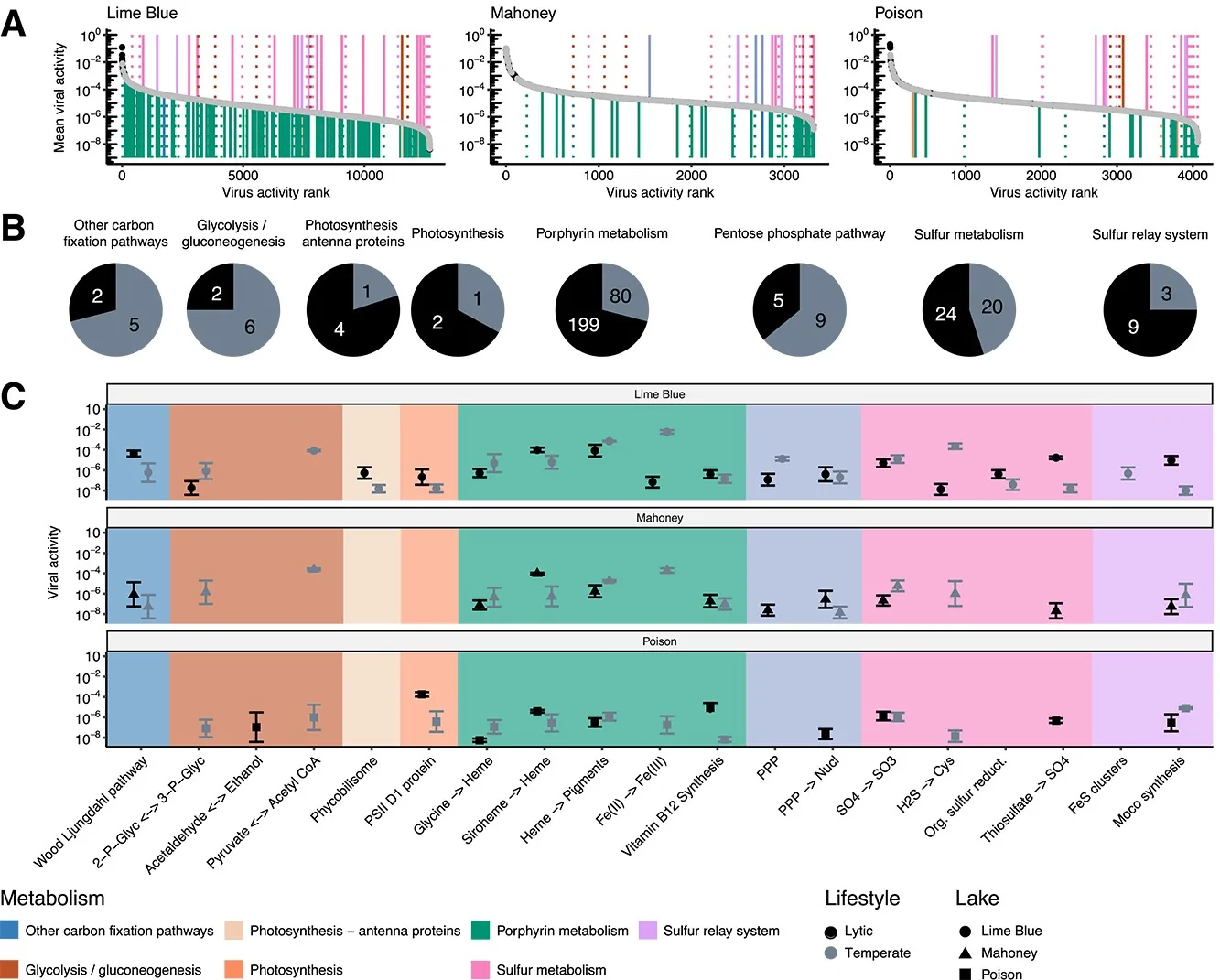
Distribution of viruses carrying metabolic genes related to biosignatures. (A) Rank-activity of lytic and temperate viruses. Vertical lines identify viruses carrying metabolic genes, their associated KEGG metabolic pathways, and lifestyle: lytic (solid) or temperate (dotted). (B) Number of putatively lytic and temperate viruses carrying metabolic genes within the pathways related to biosignatures across all lakes. (C) Summed activity of all viruses carrying genes involved in metabolic reactions or pathways that could influence biosignatures. Prior to summation of genes within a pathway, activity was log(*a* + *x*)-transformed, where *a* is the lowest, nonzero activity level for any one gene across all lakes divided by 10 (*a* = 3.76 × 10^−9^) and *x* is the activity of a gene. Genes are grouped by the KEGG metabolisms assigned to their functions. A list of specific genes associated with each pathway or reaction is provided in Supplementary Table S8. Values in panel A are displayed as means across replicates of each sample type, and values in panel C are displayed as means and standard errors.

The analysis of viral-encoded metabolic genes focused on those belonging to Kyoto Encyclopedia of Genes and Genomes (KEGG) metabolic pathways most likely to directly affect biosignature and biomarker production, including carbon fixation, glycolysis and gluconeogenesis, photosynthesis, antenna proteins, porphyrin metabolism, sulfur metabolism, and the sulfur relay system. Both the number of active viruses and the subset of those viruses encoding predicted metabolic genes of interest, herein referred to as metabolic genes, were higher in the Lime Blue microbial plate (active viruses *n* = 12 704; active viruses with metabolic genes *n* = 247) compared to Mahoney (active viruses *n* = 3319; active viruses with metabolic genes *n* = 84) or Poison (active viruses *n* = 4066; active viruses with metabolic genes *n* = 65, Fig. 4A). In Mahoney and Poison Lakes, ~50% and 51% (*n* = 42, *n* = 33), respectively, of the viruses carrying these metabolic genes were temperate, whereas in Lime Blue, only 31% (*n* = 76) were temperate. Most of the genes in pathways of interest were encoded by lytic viruses, except for “other carbon fixation pathways,” “glycolysis/gluconeogenesis,” and “pentose phosphate pathway,” which were more often found in temperate viruses (Fig. 4B). Genes related to sulfur metabolism showed a near 50% split between viral lifestyles (Fig. 4B). Lytic viruses carrying metabolic genes related to thiosulfate oxidation and molybdenum cofactor synthesis were more active in Lime Blue, whereas in Poison Lake, lytic viruses carried vitamin B12 synthesis genes (Tukey’s test, *P*-value <.05, Fig. 4C). Lime Blue temperate viruses carrying genes involved in the pentose phosphate pathway, incorporation of sulfide into cysteine, iron storage, and iron cluster assembly were more active than lytic viruses (Tukey’s test, *P*-value <.05, Fig. 4C). In Poison Lake, temperate viruses with genes related to the conversion of glycine to heme (and likely pigment production) were more active than lytic viruses, whereas in Mahoney Lake, temperate viruses with genes involved in the reduction of sulfate to sulfite were more active than their lytic counterparts (Tukey’s test, *P*-value <.05, Fig. 4C).

**Figure 5:**
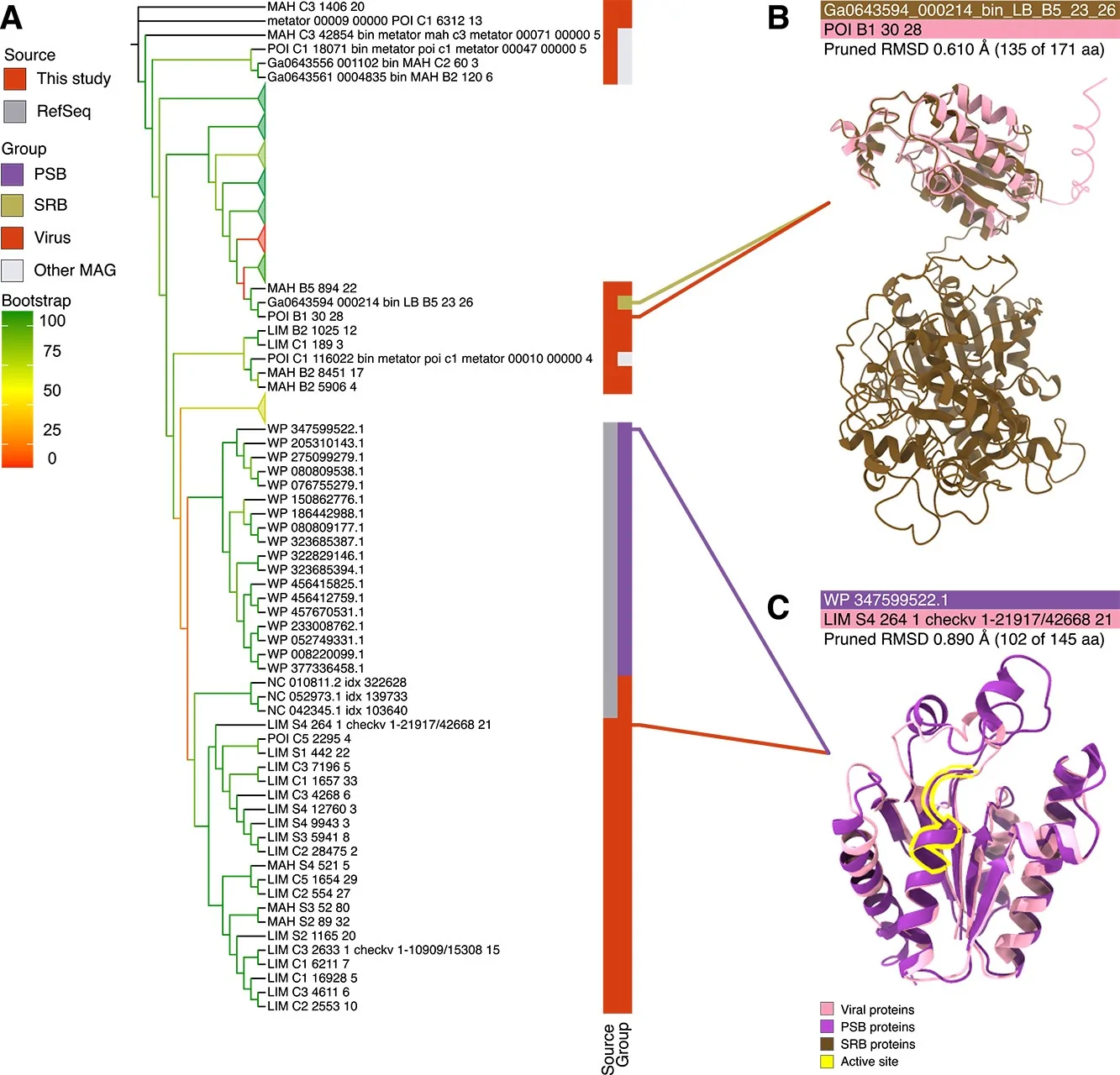
Phylogenetic and structural comparisons of viral *cysC* amino acid sequences. A) Maximum likelihood phylogenies of viral, MAG, and PSB, GSB, or SRB RefSeq csyC amino acid sequences. “Source” indicates whether the sequence was obtained from metagenomes/metatranscriptomes in this study or the RefSeq database. “Group” indicates if the sequence is derived from a virus, PSB, GSB, SRB, or another MAG. Branches represent the bootstrap value. (B, C) Comparisons of AlphaFold structures of viral *cysC* proteins to their closest SRB (B) or PSB (C) homolog. The active site of the PSB *cysC* enzyme is highlighted.

Phylogenetic and structural comparisons were performed on the gene *cysC*, which encodes adenylylsulfate kinase, an enzyme that converts adenylyl sulfate (APS) to 3′-phosphoadenylyl sulfate (PAPS), an intermediate in the reduction of sulfate to sulfite, and therefore, is central to sulfur cycling in this system. The amino acid sequences of viral-encoded *cysC* were compared with reference sequences and sequences derived from PSB, GSB, and SRB MAGs in our dataset. Two viral *cysC* proteins from Mahoney and Poison Lakes clustered with the SRB MAG *Desulfomonilales* (Fig. 5A). Structural comparison revealed a significant similarity between the *cysC* domain of the bifunctional *cysNC* protein encoded by the SRB and one of the viral *cysC* proteins (RMSD = 0.610; Fig. 5B). A majority of the viral cysC proteins from this study (62%) grouped within a clade of *Pseudomonadota*-infecting phages and had low bootstrap support (23/100) with a clade of PSB reference sequences (Fig. 5A). Structural comparison of the most closely related viral and PSB-derived *cysC* proteins revealed high similarity, with a root-mean-square deviation of 0.890 Å across 102 of 145 amino acids (Fig. 5C). Similar phylogenetic and structural comparisons were conducted for *cysK*, which is involved in the incorporation of sulfide into L-cysteine, and *bfr*, which is involved in Fe (II) storage. Both trees had viral representatives that clustered with SRB homologs and showed significant structural similarity (RMSD <1 Å; Supplementary Figs. S4 and S5). Additionally, two viral *cysK* genes were structurally similar to GSB reference proteins and two viral *bfr* proteins were highly similar to PSB reference proteins (RMSD <1 Å; Supplementary Figs S4 and S5).

## Discussion

### Decoupling between abundance and activity in phototrophic sulfur bacteria

Understanding the biotic and abiotic factors that control bacterial metabolism and, therefore, the production and expression of biosignatures remains a major challenge with implications for geobiology, global biogeochemistry, and astrobiology. PSB and GSB produce diagnostic pigments and isotopic fractionations that are widely used to reconstruct anoxic photic zones through Earth’s history, yet the controls on these signatures remain poorly constrained in modern analogs of ancient oceans [5, 28, 29]. Here, we provide evidence that variability in biosignature expression can arise from a decoupling between bacterial abundance and activity. Across the microbial plates of three study lakes, PSB and GSB exhibited contrasting relationships between their relative abundances in the microbial community and their activities (Fig. 1B). PSBs consistently showed activity exceeding expectations based on their abundance across both low- and high-sulfide chemoclines. In contrast, GSBs were comparatively less active in Lime Blue, where they were the dominant phototrophs, and in Poison Lake. Higher relative GSB activity occurred only in Mahoney Lake, where they accounted for <0.001% of the community, likely below our approach’s ability to detect true differences. Together, these observations indicate that sulfide availability alone does not explain the observed decoupling between abundance and activity in PSB and GSB on microbial plates.

### Contrasting viral interactions in purple sulfur bacteria and green sulfur bacteria

The elevated activity of PSBs relative to their abundance occurred concurrently with several indicators of lysogenic phage infections, suggesting that lysogeny could contribute to sustaining high PSB activity. First, temperate viruses were more abundant and active in the microbial plates of lakes dominated by PSB than in Lime Blue Lake, where GSB dominates, and sulfide concentration is orders of magnitude lower (Fig. 2). Second, most temperate viruses had low coverage of transcripts across the genome, indicating the expression of few genes (such as repressors) during lysogeny, and only a few of these viruses displayed high activity levels with high coverage across the genome, which would be expected from temperate viruses undergoing the lytic cycle (Supplementary Fig. S6). Third, all virus–host associations identified for PSBs in the dataset are exclusively with temperate viruses (Fig. 3), and the VHRs of abundance and activity of these virus–host pairs were low, which may indicate limited activity and replication relative to the host, consistent with a temperate lifestyle. Although the high-throughput viral identification methods applied here did not identify a medium- or high-quality prophage integrated into the genomes of the dominant PSB genus *Thiohalocapsa*, likely due to the fragmented nature of MAGs [86], manual inspection of this genome using VirSorter2 identified two putative prophage regions, each containing at least two viral genes (Supplementary Table S9). Taken together, these observations indicate that PSBs within the microbial plate may be subject to weak top–down viral control and that lysogeny may be the predominant viral strategy associated with these populations. Lysogeny could promote higher host activity through a Make-the-Winner regime in which superinfection exclusion increases host fitness [87, 88].

In contrast to PSBs, the comparatively low activity of GSB populations coincided with indicators of stronger lytic top–down viral control. The first line of evidence supporting this conclusion is the lower abundances of temperate viruses in the microbial plate of Lime Blue (dominated by GSB and with trace sulfide concentrations) than in samples dominated by PSB and with larger sulfide pools (Fig. 2). Second, dominant GSB taxa, such as *Chlorobium*, were only associated with lytic viruses (Fig. 3). Third, total viral abundances and virus-to-microbe ratios in the Lime Blue microbial plate, quantified via epifluorescence microscopy, were significantly higher than those in surface waters and microbial plates of Mahoney and Poison Lakes (Supplementary Fig. S7, Supplementary Table S10) [18]. Finally, bacteria containing CRISPR-Cas defense systems were more abundant and active in Lime Blue Lake, including the most dominant GSB, whereas the dominant PSB in lakes with a large sulfide pool only carried RM systems (Fig. 2). Bacteria infected by lytic viruses can use both CRISPR-Cas and RM systems, whereas RM systems are most effective against temperate viruses due to the risk of autoimmunity via CRISPR after integration [89]. We speculate that the reduced activity of GSB relative to their abundance could therefore result from lytic infections redirecting host resources and transcription toward virion production and removing active cells from the population [42, 90–92]. These dynamics contrast with the evidence for lysogeny in PSB populations, even when both guilds co-occur within the same chemocline. A caveat to the conclusion that GSBs are under lytic top–down control is the relatively low VHRs calculated from abundance and activity of the dominant *Chlorobium* virus–host pair (Fig. 3). However, this observation may be explained by Kill-the-Winner cycles [93] or simply by a fraction of the GSB population being infected at any given time, considering their very large population size. Additional work on this specific pair is necessary to address this open question.

### Infection strategies are host-linked

The consistently high PSB activity relative to their abundance and their association with temperate viral infections across all three microbial plates with wide ranges of sulfide availability (0.006–25.389 mM) suggest that lysogeny is favored by host traits rather than by external geochemical conditions. One such trait may be the host’s genome architecture. Lysogeny has been shown to be more prevalent in hosts with larger genomes, which provide increased availability of neutral integration sites, greater regulatory complexity, and enhanced buffering against the metabolic costs of prophage maintenance [94–96]. Consistent with this framework, the average genome length of PSBs within *Chromatiaceae* (~4.31 Mb) is ~1.75x larger than that of GSBs within *Chlorobi* (~2.47 Mb), potentially increasing PSB susceptibility to stable prophage integration. Differences in antiviral defense strategies further support this interpretation. PSB MAGs with RM systems showed a higher prevalence and activity (Fig. 2C and D). The most abundant and active GSB MAGs carried CRISPR-Cas systems, indicating lytic pressure. These contrasting defense architectures could also explain the observed divergence in viral infection strategies between PSBs and GSBs.

### Sulfate-reducing bacteria and viral regulation of sulfur cycling within microbial plates

**Figure 6:**
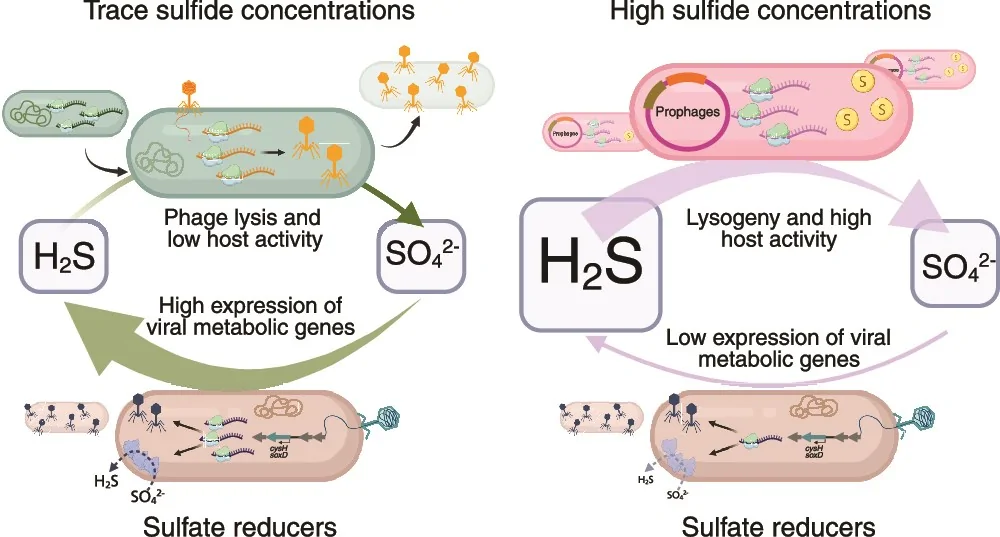
Conceptual framework of contrasting viral controls of phototrophs and sulfur regeneration in meromictic lakes. In systems with trace sulfide concentrations (left), lytic top–down controls on GSBs reduce host activity and redirect transcription to viral propagation, limiting the amount of sulfide oxidized. Viruses carry and transcribe more genes related to sulfide regeneration, helping to sustain the limited pool of sulfide available (bottom, left). In systems with higher sulfide availability (right), integrated viruses (prophages, higlighted DNA segments) protect the host from lytic infections and promote photosynthetic activity and sulfide oxidation. Activity of SRB and viruses carrying genes related to sulfur regeneration is limited and contributes little to the sulfide pool (bottom, left).

Beyond their role in structuring phototrophic populations, viruses appear to modulate sulfur cycling through interactions with SRB [97]. The distribution, transcriptional level, and predicted protein structures of viral metabolic genes support a role for viruses in sustaining sulfur cycling within microbial plates, particularly under low sulfide availability. In Lime Blue Lake (0.006 mM sulfide), viruses carrying sulfur-related metabolic genes were disproportionately represented among the most active viruses compared to Mahoney (25.389 mM) and Poison (0.831 mM) lakes. Genes in Sulfur-associated pathways exhibited higher transcriptional levels in Lime Blue Lake than in the lakes with higher sulfide concentrations (Fig. 4). These genes primarily encode enzymes participating in sulfate reduction, organic sulfur reduction, thiosulfate oxidation, and sulfur relay systems, indicating a functional emphasis on sulfur regeneration rather than phototrophic metabolism (Fig. 4). This pattern contrasts with Mahoney and Poison Lakes, where the aggregated activity of viral metabolic genes associated with photosynthesis and glycolysis/gluconeogenesis was significantly higher compared to Lime Blue (Tukey’s test *P*-value <.05, Fig. 4, Supplementary Table S11). Together, these patterns suggest that in environments where sulfide availability can sustain high rates of anoxygenic photosynthesis, viruses may promote host activity through lysogeny, superinfection exclusion, and phototrophic metabolism. In contrast, in systems characterized by lower sulfide availability, lytic top–down control may limit GSB activity, and viral metabolic genes promoting the regeneration of reduced sulfur are selected (Fig. 6).

In meromictic lakes, anoxygenic phototrophs are sustained by sulfide supplied both by upward diffusion from the sulfidic monimolimnion and by *in situ* sulfate reduction by SRB within or below the microbial plate [20, 30, 98]. Even though sulfate reduction contributes to local sulfide production, the observation that SRB activity is, on average, approximately one order of magnitude lower than that of PSBs and GSBs (Fig. 1) suggests that sulfate reduction alone is insufficient to meet phototrophic sulfide demand in such systems. Within this flux-limited framework, the elevated activity of sulfide-regenerating viral metabolic genes (such as sulfate, thiosulfate, and organic sulfur reduction) in Lime Blue Lake suggests that viral infections may expand the number of pathways that reduce sulfur without substantially increasing overall SRB activity or biomass (Fig. 4C). Such viral acceleration of sulfur metabolism could promote rapid sulfur turnover while preventing sulfide accumulation, particularly in systems where sulfide is immediately consumed by highly active phototrophic sulfur bacteria (Fig. 6). This tight coupling between sulfate reduction and sulfide oxidation is consistent with models of cryptic sulfur cycling, in which reduced sulfur is continuously regenerated and consumed on short timescales, masking sulfate reduction at the level of bulk geochemistry despite high metabolic activity [20, 30, 99].

Taken together, our observations indicate that viruses may play a previously underappreciated role in regulating sulfur fluxes within microbial plates by modulating the efficiency and turnover of sulfur cycling pathways. In sulfide-limited systems, such as at the microbial plate in Lime Blue Lake, viral regulation of SRB may therefore help maintain anoxygenic primary production by buffering sulfur cycling against geochemical limitation.

The multiple lines of evidence presented here indicate a role for viral infections in the metabolism of sulfur bacteria, but these observations do not exclude the possibility that intrinsic differences between PSB and GSB, or other biotic or abiotic factors, may contribute to the decoupling in their abundance and activity. Even though our data cannot determine causal relationships, future studies should focus on resolving these interactions, including efforts to isolate GSB and PSB viruses, as well as the application of methods for identifying the activity levels of cells or viruses within an environmental sample, such as quantitative stable isotope probing or the use of fluorescence indicators of activity paired with cell sorting [100, 101].

### Predictions for the geological record

In PSBs, transcriptional activity exceeds abundance, indicating elevated metabolic rates relative to population size, including increased photosynthetic rates via the synthesis of light-harvesting compounds such as lipid pigments [11, 102–104]. Some of these compounds are recalcitrant, notably the lipid biomarker okenone, which is widely used as a diagnostic indicator of PSB and has been detected in Proterozoic (1.64 billion years) sedimentary rocks [105]. Therefore, viral modulation of PSB metabolism has the potential to increase the production of PSB-derived biomarkers, thereby expanding the biomarker pool and increasing the likelihood that these compounds are preserved in the geological record.

Enhanced metabolic activity may also influence sulfur isotope systematics associated with PSB-dominated sulfur cycling. Higher metabolic rates are generally associated with reduced net isotopic fractionation between substrates and products, as rapid intracellular processing limits isotopic equilibration and isotopic “sorting” between light (^32^S) and heavy (^34^S) isotopes. Microbial sulfide oxidation is generally associated with small sulfur isotopic fractionations and thus often exerts only a limited isotopic overprint on bulk sulfate–sulfide systematics [106]. Notable exceptions exist; for example, sulfide oxidation can, in some cases, generate substantial ^34^S enrichment in the produced sulfate (+12.5‰) [107]. In the framework hypothesized here, in which PSB activity is increased by temperate viral infection, we would predict a lower apparent sulfur isotopic fractionation between sulfate and sulfide (Δ^34^S = δ^34^S_sulfate_ − δ^34^S_sulfide_). In this framework, limited top–down control could simultaneously enhance the accumulation of PSB-derived biomarkers while dampening the expression of characteristic bulk sulfur isotope fractionation. In contrast, our proposed framework with strong lytic viral control of GSBs is expected to reduce overall activity, diminishing pigment production, and weakening the contribution of GSB-derived biomarkers to the sedimentary record.

The observations of elevated expression of sulfate reduction genes in both low- and high-sulfide environments, together with a large number of virus–host associations (*n* = 84), suggest that viral infection may also modulate SRB. SRB preferentially reduce ^32^S_sulfate_, and the magnitude of sulfur isotope fractionation expressed during sulfate reduction depends on intracellular sulfate reduction rates, as well as extracellular sulfate concentrations [108, 109]. In general, sulfur isotope fractionation is negatively related to sulfate reduction rates, whereas low intracellular rates can approach near-thermodynamic fractionation even at micromolar sulfate concentrations [110]. Viral sulfate-reducing proteins with significant structural homology to those encoded by sulfur bacteria (Fig. 5) indicate the potential for viral infections to increase sulfate reduction rates and reduce the net sulfur isotope fractionation between sulfate and sulfide at the bulk scale. Rather than increasing sulfide accumulation, such viral acceleration of sulfate reduction could be expected to increase the sulfide flux through the system, thereby reinforcing rapid sulfur turnover. The presumed viral-enhanced regeneration of reduced sulfur would increase substrate availability for sulfide-oxidizing phototrophs, thereby further coupling reductive and oxidative sulfur cycling within microbial plates.

On early Earth, viral stimulation of sulfate reduction could have contributed to locally or episodically elevated sulfide fluxes in photic zones. Enhanced sulfide availability would have acted as a sink for molecular oxygen and intensified competition for light and nutrients between anoxygenic phototrophs and oxygenic cyanobacteria [1, 111]. Even though viral regulation alone is unlikely to account for global redox states, such biologically mediated feedback could have contributed to maintaining heterogeneity and suppressing net oxygen accumulation at regional scales. Over geological timescales, the cumulative effects of these processes may have contributed to the prolonged delay between the emergence of oxygenic photosynthesis as early as the Eoarchean (~3.5 Ga) [112, 113] and the oxygenation of Earth’s atmosphere (>2.5 Ga) [114], as well as the much later widespread oxygenation of the ocean water column (>635 Ma) [115, 116].

## Conclusion

Within meromictic lakes, microbial plates formed by anoxygenic phototrophs represent hotspots of intense microbial activity and tight biogeochemical coupling. By integrating metagenomics, metatranscriptomics, and virus–host linkage approaches, we show that viral infection strategies in these microbial plates are structured primarily by functional guilds (PSB, GSB, and SRB) rather than by lake-scale geochemical differences. A central conclusion of this work is that microbial abundance is not a direct proxy for metabolic activity within microbial plates of anoxic and sulfidic environments. PSBs consistently exhibit activity that exceeds their abundance and are linked exclusively to temperate viruses. In contrast, GSBs display comparatively high abundance but reduced activity and are predominantly associated with lytic viruses. Beyond phototrophs, SRB are associated with both lytic and temperate viruses encoding genes implicated in sulfur transformations, with sulfur-regenerating functions particularly enriched among the active viruses in the sulfide-limited Lime Blue microbial plate.

## Supplementary Material

Supplementary_material_wrag197

## Data & code availability

Transcriptomic and Hi-C sequence data have been deposited to the National Center for Biotechnology Sequence Read Archive (NCBI SRA) under accession code PRJNA1161448. Metagenomes and MAGs have been published by the Joint Genome Institute and can be accessed using the DOI 10.46936/10.25585/60008509. Detailed description of the bioinformatics pipelines, data tables, and R code for data wrangling and visualization can be found at https://github.com/Silveira-Lab/SPACE2023_lifestyles.
