# Supplementary material for: Abundance–activity decoupling in sulfur-cycling bacteria reflects viral infection types in meromictic lakes
Source: ISME J. 2026 Jul 31;20(1):wrag197. doi: 10.1093/ismejo/wrag197 (PMC13584214; doi:10.1093/ismejo/wrag197)
Supplement: Supplementary file 1 [file supplementary_material_wrag197.zip › Supplementary_Figures_final_wrag197.docx]

Supplementary Figure 1. Distribution of anoxic phototrophs and sulfate reducers across all samples. A-B) Abundance (darker colors) and activity (lighter colors) of anoxygenic phototrophs (A) and sulfate reducers (B) in all samples. Anoxic phototrophs are grouped as either PSBs (pink, purple), GSBs (light and dark green), or aerobic anoxic phototrophs (white, gray). Sulfate reducers are colored according to the depth of the sample taken surface (light and dark teal), plate (pink and purple), and bottom (tan and gold). Results of Tukey’s tests comparing abundance to transcription are denoted by ns = not significant, * = 0.05, ** = 0.01, *** = 0.001, **** = 0.0001.

Supplementary Figure 2. Distribution of different viral lifestyles and MAGs containing phage defense system across all samples. (A) Putatively lytic vOTUs, (B). temperate vOTUs including prophages, (C) MAGs with genes annotated as Cas systems, and (D) MAGs with genes annotated as restriction modification systems. Activity is indicated by lighter colors and abundance by darker colors. The colors of bars represent the depth of the sample taken surface (light and dark teal), plate (pink and purple), and bottom (tan and gold). Results of Tukey’s tests comparing abundance to transcription are denoted by ns = not significant, * = 0.05, ** = 0.01, *** = 0.001, **** = 0.0001.

Supplementary Figure 3. Virus-host interaction network. (A) Visualization of interaction between viruses and hosts. Host (circles) are colored according to their class. Phages are indicated by arrows and prophage have a black boarder. (B) Percentage of viruses identified using the four different methods including Hi-C and *in silico* approaches. (C) Distribution of the numbers of connections made by lake and sample depth. Bars are separated and colored according to the host class. The three columns in on the left reflect only *in silico* connections while the 2 columns on the right represent only Hi-C connections. (D) Connections made between original viral contigs (left) assembled from a specific sample type and the sample type that the original MAG (right) was assembled and binned from. Colors indicate the source sample type of the original viral contig.

Supplementary Figure 4. Phylogenetic and structural comparisons of viral cysK genes. A) Maximum likelihood phylogenies of viral, MAG, and PSB, GSB, or SRB RefSeq csyK genes. Source indicates whether the sequence was gathered from metagenomes/metatranscriptomes in this study (red) or the RefSeq database (gray). Group indicates if the sequence is derived from a virus (red), PSB (purple), GSB (green), SRB (yellow), or a MAG (white) not included in these groups. Branches are colored according to bootstrap value with green indicating 100% and red 0%. B-C) Comparisons of Alphafold structures of viral cysK genes to their closet SRB (B) or GSB (C) homolog. Viral proteins are colored in pink, SRB in brown, and GSB in green. The catalytic residues of the SRB and GSB proteins are indicated by the label CR.

Supplementary Figure 5. Phylogenetic and structural comparisons of viral bfr genes. A) Maximum likelihood phylogenies of viral, MAG, and PSB, GSB, or SRB RefSeq bfr genes. Source indicates whether the sequence was gathered from metagenomes/metatranscriptomes in this study (red) or the RefSeq database (gray). Group indicates if the sequence is derived from a virus (red), PSB (purple), GSB (green), SRB (yellow), or a MAG (white) not included in these groups. Branches are colored according to bootstrap value with green indicating 100% and red 0%. B-C) Comparisons of Alphafold structures of viral bfr genes to their closet PSB (B) or SRB (C) homolog. Viral proteins are colored in pink, PSB in purple, and SRB in brown.

Supplementary Figure 6. Breadth of coverage of temperate viruses in the bacterial plates of each lake. A) Abundance and B) activity levels of viruses grouped by the read or transcript coverage across the viral contig. The number of viruses within each coverage group is indicated above the bars by n.

Supplementary Figure 7. Microscopy counts of the bacterial plates of each lake. The panels show the results of counts from epifluorescence microscopy for bacterial cells per mL, viruses per mL, and the virus-to-microbe ratio (VMR). Wilcox rank test p-values are indicated by the bars on the right of each panel (ns = not significant, * = 0.05, ** = 0.01, *** = 0.001, **** = 0.0001).
